# Facilitators of Person-Centered Dementia Care Strategies: Insights From Staff and Leadership

**DOI:** 10.1093/geroni/igaf122.414

**Published:** 2025-12-31

**Authors:** Yoon Chung Kim, Nancy Kusmaul, Sarah Holmes, Michael Lepore, Jing Wang, Laura Davie, Briana Murray, Kirsten Corazzini

**Affiliations:** University of Maryland School of Medicine, Baltimore, Maryland, United States; University of Maryland, Baltimore County, Baltimore, Maryland, United States; University of Maryland School of Nursing, Baltimore, Maryland, United States; University of Massachusetts Amherst, Amherst, Massachusetts, United States; University of New Hampshire, Durham, New Hampshire, United States; University of New Hampshire, Durham, New Hampshire, United States; University of Massachusetts Amherst, Amherst, Massachusetts, United States; University of New Hampshire, Durham, New Hampshire, United States

## Abstract

Person-centered dementia care (PCDC) strategies improve the well-being of residents living with dementia (RLWD) in long-term care (LTC). We explored facilitators to PCDC strategies in low-resource LTC settings. Semi-structured interviews occurred June 2023 - February 2024 to explore stakeholder perspectives in low-resource settings. Participating facilities were two nursing homes and two assisted livings in US Census Bureau-designated medically underserved areas. Interviews were audio recorded and transcribed using NVivo 14.0. Coauthors coded the interviews guided by the Values, Individualized approach, Perspective of PLWD, and Social environment model. Participants were 20 direct care staff and 7 administrators, with between 3 months and 27 years’ experience. Facilitators of PCDC were identified at multiple levels, highlighting opportunities to strengthen supportive practices. Organizational facilitators included adequate staffing, information sharing systems, regular dementia education, positive work culture, staff empowerment, and flexibility in care decisions. Interpersonal facilitators based on the LTC setting social environment included strong teamwork, inter-staff communication, good relationships with care partners, and trust/rapport with residents. Individual facilitators were based on individual staff values associated with delivering PCDC, e.g., positive attitudes about care work and motivation for additional dementia education. Given the well-documented benefits of PCDC, identifying and cultivating these facilitators, particularly in resource-limited settings, is critical to enhancing the well-being of RLWD. Although one identified facilitator is adequate staffing, low-resource settings can support PCDC through effective data sharing, regular training, and fostering teamwork between staff, RLWD, and care partners. Future research should identify factors that foster employees’ positive attitudes and motivation for additional training.

